# Efficacy of fluoxetine for anorexia nervosa caused by chemotherapy in patients with cholangiocarcinoma

**DOI:** 10.1097/MD.0000000000015945

**Published:** 2019-06-14

**Authors:** Lin-Qi Guo, Hua-Wei Sun, Chun-Ye Zhang, Yao Feng, Xin-Li Teng, Yi-Kun Qu

**Affiliations:** a Second Ward of Surgery Department; b Department of Chemotherapy and Radiotherapy; c Department of Emergency; d Department of Chinese Medicine, First Affiliated Hospital of Jiamusi University, Jiamusi; e Department of Chemotherapy and Radiotherapy, Jiamusi Cancer Hospital, China.

**Keywords:** anorexia nervosa, chemotherapy, cholangiocarcinoma, fluoxetine

## Abstract

**Background:**

Fluoxetine has been reported to treat anorexia nervosa (AN) caused by chemotherapy in patients with cholangiocarcinoma effectively. However, no study systematically investigated its efficacy and safety. Thus, this study will systematically assess its efficacy and safety for AN caused by chemotherapy in patients with cholangiocarcinoma.

**Methods:**

A comprehensive literature search for relevant studies will be conducted from the following databases from inception to the present: MEDILINE, EMBASE, Cochrane Library, Web of Science, PSYCINFO, Allied and Complementary Medicine Database, Chinese Biomedical Literature Database, and China National Knowledge Infrastructure. All randomized controlled trials on assessing the efficacy and safety of fluoxetine for AN caused by chemotherapy in patients with cholangiocarcinoma will be considered for inclusion in this study. RevMan V.5.3 software will be used for risk of bias assessment and statistical analysis.

**Results:**

This study will summarize the latest evidence of fluoxetine for AN caused by chemotherapy in patients with cholangiocarcinoma through assessing outcomes of weight, depression, anxiety, and quality of life. Additionally, any adverse events will also be analyzed.

**Conclusion:**

The findings of this study will provide most recent evidence of fluoxetine for AN caused by chemotherapy in patients with cholangiocarcinoma.

**Systematic review registration:**

PROSPERO CRD42019131583.

## 1 Introduction

Cholangiocarcinoma is a primary liver cancer with features of cholangiocyte differentiation.^[1,2]^ It originates from the cholangiocytes lining the biliary tree.^[3,4]^ It is also the secondary common liver cancer, and contributes to more than 10% of all liver cancers.^[5]^ Additionally, its incidence has steadily increased worldwide.^[6,7]^ Patients experience such disorder often manifest as yellow skin and eyes (jaundice), intensely itchy skin, and stool that is white in color.^[8,9]^

Several managements are available for this disorder, including surgery, radiotherapy, chemotherapy, and supportive care.^[10–16]^ Although these managements are effective for patients with chemotherapy, they also suffer from a variety of adverse events, such as anorexia nervosa (AN), fatigue, hair loss, infection, anemia, nausea and vomiting, as well as constipation,^[17–19]^ especially for AN. If it cannot be treated very well, it may greatly affect the efficacy of cholangiocarcinoma treatment, and quality of life for the patients.

Numerous studies have reported that fluoxetine can be used for AN treatment caused by chemotherapy in patients with cholangiocarcinoma.^[20–22]^ However, no study has systematically assessed the efficacy and safety of fluoxetine for AN caused by chemotherapy in patients with cholangiocarcinoma. Therefore, this study will systematically evaluate the efficacy and safety of fluoxetine for AN caused by chemotherapy in patients with cholangiocarcinoma.

## 2 Methods

### 2.1 Ethics and dissemination

This study does not need ethic approval because no individual patient data will be used. The results of this study will be published in peer-reviewed journals.

### 2.2 Study registration

This study has been registered on PROSPERO CRD42019131583. We have reported all information in accordance with the guideline of preferred reporting items for systematic reviews and meta-analysis (PRISMA) protocol statement.^[23]^

### 2.3 Eligibility criteria for study selection

#### 2.3.1 Type of studies

All randomized controlled trials (RCTs) on assessing the efficacy and safety of fluoxetine for AN caused by chemotherapy in patients with cholangiocarcinoma will be included. However, nonclinical studies, non-RCTs will not be considered in this study.

#### 2.3.2 Type of participants

Any cholangiocarcinoma patients with AN caused by chemotherapy will be considered for inclusion without any restrictions of race, age, gender, and economic status.

#### 2.3.3 Type of interventions

Experimental group: patients received fluoxetine monotherapy only for AN.

Control group: patients received any therapies except any forms of fluoxetine.

#### 2.3.4 Type of outcome measurements

Primary outcome includes change of weight. Secondary outcomes consist of depression, as measured by Beck Depression Inventory or other related scales; anxiety, as assessed by the Beck Anxiety Inventory or other related tools; and quality of life, as evaluated by Yale Brown Cornell Obsessive Compulsive Scale for Eating Disorders or other relevant scales, as well as any adverse events.

### 2.4 Data sources and search strategy

We will conduct a comprehensive literature search for relevant studies from the following databases from inception to the present: MEDILINE, EMBASE, Cochrane Library, Web of Science, PSYCINFO, Allied and Complementary Medicine Database, Chinese Biomedical Literature Database, and China National Knowledge Infrastructure without any language restrictions. In addition, clinical registry and reference lists of included studies will also be searched to avoid missing any qualified studies. The sample of the search strategy for MEDLINE is presented in Table 1. In addition, we will also apply similar search strategy to any other databases.

**Table 1:**
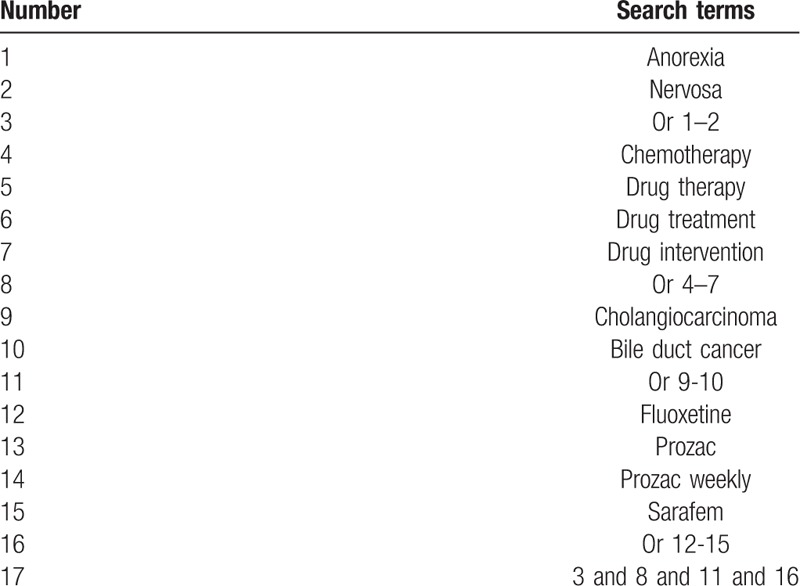
Search strategy for MEDLINE.

### 2.5 Data collection and management

#### 2.5.1 Study selection

All database records will be imported into Endnote 7.0 software, and duplicated literatures will be removed. Two authors will review the titles and abstracts for all literatures independently, and all irrelevant studies will be excluded. After that, all remaining records will be read by full texts to determine if they meet the final eligibility criteria. If any disagreements occur, a third author will help to resolve through discussion. The results of the study selection will be summarized in the PRISMA flow diagram.

#### 2.5.2 Data extraction

Two authors will extract all important information independently according to the predefined data extraction form. Any divergences will be resolved by a third author through discussion. We will extract all general information of studies and patients, study methods, treatment details, as well as outcomes and safety. Whenever there is insufficient or missing data, we will contact primary authors for request. If we cannot receive those data, we will just analyze available data. Moreover, we will discuss its possible impacts in the text.

### 2.6 Quality assessment for eligible studies

We will use RevMan 5.3 software to assess the methodological quality for all eligible studies according to the standards of Cochrane risk of bias tool. This tool includes 7 parts for evaluation, and each part can be judged as high risk of bias, unclear risk of bias or low risk of bias. Two independent authors will assess methodological quality for each eligible study. A third author will be invited to solve the issues if any disagreements will occur between 2 authors.

### 2.7 Statistical analysis

RevMan V.5.3 software will be used to conduct statistical analysis in this study. *I*^2^ statistic test will be used to check heterogeneity among included studies. *I*^2^ ≤ 50% indicates low heterogeneity, and we will use a fixed-effect model to pool the data, and to conduct meta-analysis. On the other hand, *I*^2^ > 50% indicates significant heterogeneity, and we will use a random-effect model to pool the data. Additionally, subgroup will also be conducted to find any possible reasons that may cause significant heterogeneity. If there is still substantial heterogeneity after subgroup analysis, we will not pool the data, and meta-analysis will not be performed. Instead, we will report a narrative summary for the outcome results.

Subgroup analysis will be carried out in accordance with the different characteristics of study, treatments, controls, and outcomes. In addition, sensitivity analysis will also be performed by removing low-quality studies. Furthermore, we will also check reporting bias by using funnel plots and Egger linear regression test if more than 10 eligible studies are included in this study.

## 3 Discussion

This study will systematically assess the efficacy and safety of fluoxetine for AN caused by chemotherapy in patients with cholangiocarcinom. We will search comprehensive databases without any language restrictions to avoid missing any potential qualified studies. The methodological quality will be assessed by Cochrane risk of bias tool. The results of this study will summarize latest evidence of fluoxetine for AN caused by chemotherapy in patients with cholangiocarcinoma. It may benefit either for clinical practice or for the patients.

## Author contributions

**Conceptualization:** Lin-Qi Guo, Hua-Wei Sun, Chun-Ye Zhang, Xin-Li Teng, Yi-Chun Qu.

**Data curation:** Lin-Qi Guo, Yao Feng, Xin-Li Teng, Yi-Chun Qu.

**Formal analysis:** Hua-Wei Sun, Chun-Ye Zhang, Yao Feng.

**Funding acquisition:** Yi-Chun Qu.

**Investigation:** Yi-Chun Qu.

**Methodology:** Lin-Qi Guo, Hua-Wei Sun, Chun-Ye Zhang, Yao Feng.

**Project administration:** Yi-Chun Qu.

**Resources:** Lin-Qi Guo, Hua-Wei Sun, Chun-Ye Zhang, Yao Feng, Xin-Li Teng.

**Software:** Lin-Qi Guo, Hua-Wei Sun, Chun-Ye Zhang, Yao Feng.

**Supervision:** Xin-Li Teng, Yi-Chun Qu.

**Validation:** Lin-Qi Guo, Yao Feng, Xin-Li Teng, Yi-Chun Qu.

**Visualization:** Lin-Qi Guo, Hua-Wei Sun, Chun-Ye Zhang, Yao Feng, Xin-Li Teng, Yi-Chun Qu.

**Writing – original draft:** Lin-Qi Guo, Hua-Wei Sun, Chun-Ye Zhang, Xin-Li Teng.

**Writing – review and editing:** Lin-Qi Guo, Hua-Wei Sun, Chun-Ye Zhang, Yao Feng, Xin-Li Teng, Yi-Chun Qu.
